# Adiponectin and Its Receptors in Chronic Hepatitis B Patients With Steatosis in China

**DOI:** 10.5812/hepatmon.6065

**Published:** 2013-04-18

**Authors:** Di Wu, Hongqi Li, Guoan Xiang, Liwei Zhang, Lihong Li, Yongmei Cao, Jinqian Zhang

**Affiliations:** 1 Department of Ultrasound, Air Force General Hospital of PLA, Beijing, China; 2 Department of Radiation Oncology, Air Force General Hospital of PLA, Beijing, China; 3 Department of General Surgery, the Second People's Hospital of Guangdong Province, Guangzhou, China; 4 Department of Cardiology, the First Affiliated Hospital of Chinese PLA General Hospital, Beijing, China; 5 Department of Neurosurgery, Tangdu Hospital, Fourth Military Medical University, Xi’an, China; 6 International Mongolian Hospital, Hohhot of Inner Mongolia, Hohhot, China; 7 Institute of Infectious Diseases, Beijing Ditan Hospital, Capital Medical University, Beijing, China; 8 Daping Hospital,Third Military Medical University, Chongqing, China

**Keywords:** Hepatitis B, Chronic, Adiponectin, Receptors, Adiponectin, liver

## Abstract

**Background:**

HBV infection is a serious public health problem worldwide, which can contribute to the incidence of chronic hepatitis B (CHB), cirrhosis, and hepatocellular carcinoma (HCC).

**Objectives:**

In the present report, we assessed the association between adiponectin, its receptors and hepatic steatosis, fibrosis, and inflammation with hepatitis B virus.

**Patients and Methods:**

Liver biopsies from 89 patients with untreated chronic hepatitis B (34 steatosis vs. 55 without steatosis) were analyzed; liver biopsies from 50 healthy adults were used as control. The liver biopsies were subjected to routine histological examination, and stained immunohistochemically for adiponectin and adiponectin receptor2 (adipoR2).

**Results:**

The two groups were found to be comparable with respect to demographic, biochemical, metabolic, histological, and viral characteristics. BMI, γ-GT, FPG, insulin, and insulin sensitivity estimated by the HOMA index were significantly higher in patients with steatosis. The viral load of HBV and HBeAg positivity was higher in patients with steatosis than those without steatosis. High serum adiponectin levels were significantly correlated with abnormal serum ALT level (vs. normal ALT, P = 0.000), and HBV genotype C (vs. genotype B, P = 0.018). In patients with chronic HBV, the insulin sensitizing adipokine adiponectin, and its receptor AdipoR2were associated with steatosis. While adiponectin may becorrelated with inflammation, adiponectin, and its receptors were not associated with viral factors.

**Conclusions:**

Our results suggest that the role of adiponectin might be impaired in chronic hepatitis B with steatosis. Reduced hepatic expression of adiponectin and adipoR2 might be of pathophysiological relevance in CHB patients with steatosis. These findings indicated that reduced liver adiponectin expression may play an important role in the pathogenesis, and progression of CHB patients with steatosis. However, hepatic expression of adiponectin, and adipoR2 was not associated with various measures of HBV infection.

## 1. Background

HBV infection is a serious public health problem worldwide, which can contribute to the incidence of chronic hepatitis B (CHB), cirrhosis, and hepatocellular carcinoma (HCC). As many as 3.5 billion individuals are infected with HBV, and are HBV carriers worldwide. A large number of clinical studies show that HBV infection is closely related to the development of diabetes, fatty liver, and other metabolic diseases. Steatosis is becoming increasingly recognized in overweight or diabetic subjects in the absence of significant alcohol use (nonalcoholicfatty liver disease; NAFLD). While simple steatosis may be benign, a proportion of patients with NAFLD develop nonalcoholic steatohepatitis (NASH) which can eventually lead to cirrhosis, and liver failure (1). Adiponectin is a recently described hormone mainly produced by adipose tissue, which has anti-inflammatory, antidiabetic, insulin-sensitizing, and antiatherosclerotic properties. In addition, adiponectin can improve hepatic insulin sensitivity, decrease lipid accumulation in macrophages, and has anti-inflammatory effects (2-5). Adiponectin exerts its effects through binding to its receptors, adipoR1, and adipoR2. AdipoR1 is expressed abundantly in skeletal muscle, while adipoR2 is predominantly expressed in the liver of mice. In addition, AdipoR1 and adipoR2 mediate increased AMP kinase, and peroxisome proliferator-activated receptor (PPAR)-α ligand activity, and fatty acid oxidation, and glucose uptake, respectively (6-10). Adiponectin, an adipocyte derived polypeptide, has been shown to alleviate steatosis, and inflammation in mice with nonalcoholic fatty liver disease (NAFLD) (9). In patients with nonalcoholic steatohepatitis, lower levels of adiponectin were associated with higher grades of hepatic steatosis and necroinflammatory activity, suggesting a pathophysiological role for this adipokine in liver disease (11). In patients with chronic HCV, adiponectin was associated with steatosis only in males, and was paradoxically increased with inflammation, and the results suggest that the role of adiponectin in chronic liver diseases may be linked to both gender and etiology (12). However, little is known about the liver expression of adiponectin, and its receptor in HBV-infected patients, and in relation to steatosis.

## 2. Objectives

To further understand the role of adiponectin in the pathogenesis of CHB patients with steatosis, we conducted a study which examined the hepatic expression of adiponectin, and its receptors in patients with CHB, and then compared patients with or without steatosis to determine whether there was any correlation between the histopathological progression of CHB, and the expression of adiponectin, and its receptors.

## 3. Patients and Methods 

### 3.1 Patient Population

The study assessed 89 patients with untreated chronic hepatitis B (CHB) who had undergone a liver biopsy at the Beijing Ditan Hospital from 2006 to 2010. 50 healthy adults were used as controls. Eligible subjects were defined as positive results for serum hepatitis B surface antigen for at least six months. All patients underwent a comprehensive history, and physical examination. Patients were excluded if they had positive results for immunoglobulin (IgM) antibody to hepatitis A virus, hepatitis E virus, antibodies to HCV by means of a second or third generation HCV enzyme-linked immunoabsorbent assay, and detectable serum HCV RNA, IgM antibody to the hepatitis B virus core antigen or antibody to the human immunodeficiency virus (HIV). Further exclusion criteria included evidence or history of any autoimmune disease; clinically significant hematologic, renal, or other metabolic diseases; decompensated liver disease; drug alcohol abuse within the previous year; or history of treatment with antiviral agents. Patients were also excluded if they had past or current alcohol use. None of the patients were diabetic or were taking medications known to affect insulin sensitivity or hepatic steatosis. Body weight, and height were measured, and body mass index (BMI) computed using the following formula: weight in Kg divided by height m^2^. The study was approved by the Ethics Committee according to the Declaration of Helsinki. Informed written consent was obtained from all subjects.

### 3.2 Biochemical Analyses

Serum (n = 89) was collected at the time of liver biopsy following an overnight fast, and stored at -80°C until use. Circulating insulin, and c-peptide were determined using the Tosoh AIA600 analyzer, 2-site immuneenzymometric assays (IEMA), (Tosoh Medics, San Francisco, USA). Insulin resistance (IR) was determined using homeostasis model of assessment (HOMA) equations (13, 14). Serum adiponectin concentrations were determined using a commercially available radioimmunoassay kit (Linco Research Inc, St Charles, MO). IR was estimated using the HOMA from fasting glucose, and insulin concentrations using the following formula: HOMA-IR= [fasting plasma insulin (mIU/l) × fasting plasma glucose (FPG) (mmol/l)]/22.5.

### 3.3 Viral Determinations of the HBV

We used commercially available kits (Abbott Laboratories, North Chicago, IL) to test serum samples for Bag, and HBeAg by enzyme immunoassay. HBV DNA was extracted from 200 uL of each plasma sample using a High Pure Viral Nucleic Acid Kit (Roche Diagnostics Applied Science, Mannheim, Germany) according to the manufacturer’s instructions. The viral titer and genotype of HBV were determined by a real-time PCR-based method that used fluorescent hybridization probes, and a Light Cycler PCR machine (Roche Diagnostics Applied Science). The lower detection limit of the qualitative assay was 500 copies/ml (15).

### 3.4. Histopathological Examination

At the time of biopsy, liver tissue was (5-6 cm) immediately frozen in liquid nitrogen, and stored at -80°C until RNA extraction was performed. The sections were analyzed by an experienced hepatopathologist (AC) who was blinded to the laboratory parameters, and clinical data. The degree of inflammation was graded according to the method of Ishak (15), and fibrosis was staged according to the method of Scheuer (16). Steatosis was graded as follows: 0 (< 5% hepatocytes affected); 1 (5-29% of hepatocytes affected); 2 (30-70% of hepatocytes affected); or 3(> 70% of hepatocytes affected) (12).

### 3.5. Immunohistochemistry (IH) for Adiponectin, and Its Receptor Adipor2

Formalin fixed paraffin embedded liver biopsies (n = 89) were subjected to immunohistochemical analysis with a polyclonal antibody to adiponectin, and its receptor adipoR2 purchased from Phoenix Pharmaceuticals (Belmont, California, USA), as previously described (11). Immunohistochemistry for adiponectin, and adipoR2 was performed on liver biopsies from thirty patients with steatosis, and thirty without steatosis. The unstained 4-5 um sections were deparaffinized with xylene, and rehydrated in graded series of ethanol. The Endogenous peroxidase activity was inhibited by 3% H_2_O_2_. A heat reduced epitope retrieval technique by microwaving slides at 92 to 98 for 15 min in 10mM citric acid buffer was used to detect adiponectin, and its receptors. The slides were incubated at 40C overnight with either goat antihuman adiponectin polyclonal antibody or rabbit antihuman adipoRII polyclonal antibody. The sections were incubated with biotinylated secondary antibody for 45 min at the room temperature. The secondary antibody used was rabbit antigoat IgG for adiponectin, and goat antirabbit IgG for adipoRII. The sections were counterstained with haematoxylin, dehydrated, and mounted permanently in medium. Finally, sections were viewed on an Olympus BX51 with Kappa camera, and analyzed with Kappa ImageBase 2.2 software (Kappa opto-electronics GmbH, Gleichen, Germany). The intensity of staining was assessed semiquantitatively, and assigned an arbitrary value of 1, 2, or 3 (representing weak, moderate, and strong staining, respectively) for each specimen.

### 3.6. Determination of mRNA Levels of Adiponectin, and its Receptors

Total RNA was extracted from frozen liver biopsies (n = 89) using Trizol reagent (Gibco, Gaithersburg, Maryland, USA), and quantified by spectrophotometry. Reverse transcription of 1 μg of RNA was performed using the Omniscript RT Kit (Qiagen, Hilden, Germany). The mRNA levels of adiponectin, and its receptors AdipoR1, AdipoR2 were assayed by real-time PCR and glyceraldehyde-3-phosphate dehydrogenase (GAPDH) as control (17). The primer sequences were designed using online software (Roche Applied Science) Universal ProbeLibrary Assay Design Center (https://www.roche-applied-science.com/servlet/RCConfigureUser?URL=StoreFramesetView&storeId=10202&catalogId=10202&langId=-1&countryId=us). The primer sequences were: adiponectin, forward 5’ GGT GAG AAG GGT GAG AAA GGA 3’, reverse5’ TTT CAC CGA TGT CTC CCT TAG 3’; adipoR1, forward 5’ CTA GGG CCT GGA TCT GCT TA 3’, reverse5’ CCG GGC TAG GTA AAA GTT GG 3’; adipoR2, forward 5’ CCA ACT GGA TGG TAC ACG AA 3’, reverse5’ AAA ATG GGC TCC AAA TCT CC 3’; GAPDH forward 5’TGC ACCACCAAC TGC TTA GC 3’, reverse 5’GGC ATG GAC TGT GGT CA TGA G 3’. Assays were performed using SYBR green. The relative concentrations of mRNAs present were determined by the relative quantity. A dilution series of positive control cDNA, and ‘no template’ controls were amplified in parallel with unknowns in each assay, and the concentration in each unknown was assessed relative quantity by comparison to controls. For each gene, the average of the duplicate assays was obtained, and normalized to the expression level of GAPDH for each sample to determine relative changes in mRNA expression.

### 3.7. Statistical Analysis

Continuous normally distributed variables were represented graphically as mean ± standard deviation of the mean (SD). For statistical comparison of quantitative data between groups, analysis of variance (ANOVA) or t-test was performed. To determine differences between groups not normally distributed, medians were compared using Kruskal-Wallis ANOVA. The χ^2^ test was used when necessary for qualitative data. The association among variables was assessed by Spearman’s nonparametric correlation. All statistical analyses were performed using SPSS software version 13.0 (SPSS Inc., Chicago, IL, USA). Statistical significance was taken at the 5% level.

## 4. Results

### 4.1. Demographic, Biochemical, Metabolic, Histological, and Viral Characteristics of HBV-Infected Patients

The demographic, biochemical, metabolic, histological, and viral characteristics of 89 patients with CHB (34 with steatosis versus 55 without steatosis), including 75 male, and 14 female patients are detailed in Table 1. AST, ALT, and C-peptide were similar in patients with or without steatosis. Serum adiponectin levels measured by radioimmunoassay were similar in patients with steatosis (7.19 ± 2.99 μg/ml), and those without steatosis (10.05 ± 3.04 μg/ml; P = 0.870). BMI ranged from 19.92 to 38.76 kg/m^2^ in patients with steatosis, and ranged from 14.48 to 29.39 kg/m^2^ in patients without steatosis. 68% of patients with steatosis, and 20% of the patients without steatosis were considered overweight (BMI > 25 kg/m^2^). Body mass index, γ-GT, FPG, insulin, and insulin sensitivity estimated by the HOMA index were significantly higher in patients with steatosis. The viral load of HBV, and HBeAg positivity were also higher in patients with steatosis than those without steatosis (P = 0.017 and P = 0.007) (Table 1).

**Table 1.** Demographic, Biochemical, Metabolic, Histological, and Viral Characteristics of Patients With HBV Infection.

	Steatosis (n = 34)	Without steatosis (n = 55)	P value
**Gender, Male/Female, %**	91/9	80/20	0.159
**Age, y, Mean ± SD**	40.59±10.40	31.42±9.68	0.846
**BMI^a^, kg/m^2^, Mean ± SD**	26.62±4.44	22.32±3.06	0.028^b^
**ALT^a^, U/L, Mean ± SD**	241.17±452.52	282.95±307.07	0.270
**AST^a^, U/L, Mean ± SD**	135.75±268.82	147.13±151.32	0.145
γ-**GT,U/L, Mean ± SD**	97.47±120.87	72.02±54.23	0.002^c^
**FPG^a^, mmol/L, Mean ± SD**	5.35±2.02	4.37±0.67	0.000^c^
**Insulin,mU/L, Mean ± SD**	11.30±4.03	8.29±2.65	0.023^b^
**C-Peptide, nmol/L, Mean ± SD**	2.06±0.77	1.86±0.71	0.704
**HOMA-IR^a^, Mean ± SD**	2.81±1.80	1.60±0.55	0.000^c^
**Adiponectin,ug/ml, Mean ± SD**	7.19±2.99	10.05±3.04	0.870
**Grade of inflammation,% with 0/1/2/3/4**	0/44/36/4/0	0/22/38/36/4	0.066
**Grade of fibrosis, % with 0/1/2/3/4**	3/44/32/15/6	2/24/42/18/14	0.291
**Grade of steatosis, % with 0/1/2/3**	0/88/12/0	55/0/0/0	0.000^c^
**HBeAg +/-, %**	56/44	27/73	0.007^c^
**Viral Genotype with B/B + C/C, %**	32/3/65	42/2/56	0.652
**Viral load, copies/ml, Mean ± SD**	8.6×10^7^±4.6×10^8^	8.0×10^6^±2.3×10^7^	0.017^b^

^a^Abbreviations: BMI, body mass index; ALT, alanine aminotransferase; AST, aspartateaminotransferase; γ-GT, γ-glutamyltranspeptidase; FPG, fasting plasma glucose; HOMA-IR, homeostasis model assessment of insulin resistance.

^b^P < 0.05

^c^P < 0.01

### 4.2. Association of Serum Adiponectin With Demographic, Metabolic, and Viral Factors

**Figure 1.**
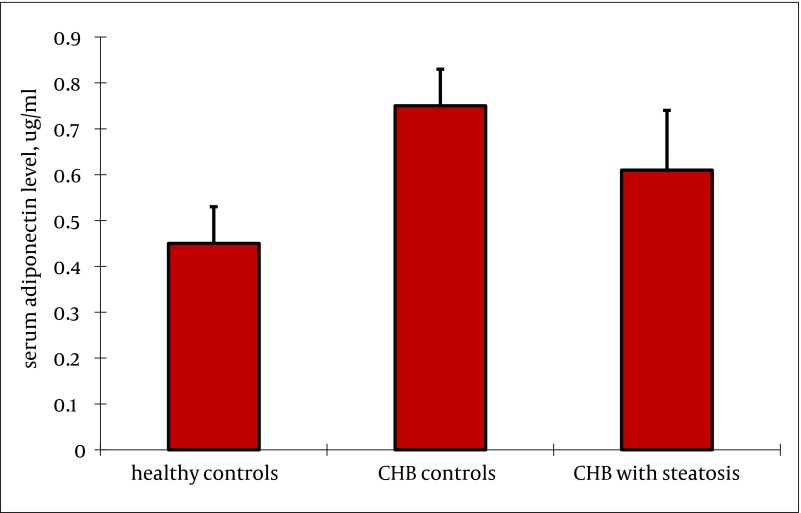
Serum Adiponectin Level in Patients With CHB (With or Without Steatosis) or Healthy Controls Serum adiponectin level was not related to gender (P = 0.180), and serum ALT level (P = 0.230) in healthy controls. Serum adiponectin level was similar between healthy adults, and CHB patients with or without steatosis. (P = 0.330).

Serum adiponectin level was not related to gender (P = 0.180) or serum ALT levels (P = 0.230) in healthy controls. Serum adiponectin level was similar between healthy adults, and patients with or without steatosis. (rs = -0.761, P = 0.330) (Figure 1). There was a significant negative correlation between serum adiponectin in patients with chronic HBV infection, and BMI (rs = -0.430, P = 0.000), FPG (rs = -0.335, P = 0.001), serum insulin (rs = -0.608, P = 0.000), serum c-peptide (rs = -0.328, P = 0.002), HOMA-IR (rs = -0.557, P = 0.000), and FFA (rs = -0.482, P = 0.000) (Table 2). But there was no significant correlation between serum adiponectin, and gender (rs = 0. 013, P = 0. 904), age (rs = -0.135, P = 0.207), ALT (rs = 0.050, P = 0.640), AST (rs = 0.044, P = 0.681), γ-GT (rs = -0.171, P = 0.108). In univariate analysis, high serum adiponectin levels were significantly correlated with abnormal serum ALT level (vs. normal ALT, P = 0.000), and HBV genotype C (vs. genotype B, P = 0.022). Viral load was available for a subset of patients, and there was no association between viral load, and serum adiponectin (rs = -0.087, P = 0.419) (Table 2).

**Table 2.** Association of Serum Adiponectin Levels With Demographic, Metabolic, Histological, and Viral Factors in Patients With Chronic HBV

Variable	rs^a^	P value
**Gender Male/Female, %**	-0.135	0.207
**Age, y**	-0.013	0.904
**BMI^a^, kg/m^2^**	-0.430	0.000^c^
**ALT^a^, U/L**	0.050	0.640
**AST^a^, U/L**	0.044	0.681
**LDH^a^, U/L**	-0.141	0.190
γ-**GT^a^, U/L**	-0.171	0.108
**FPG^a^, mmol/L**	-0.335	0.001
**FINS^a^, mU/L**	-0.608	0.000^c^
**C-Peptide, nmol/L**	-0.328	0.002^c^
**HOMA-IR^a^**	-0.557	0.000^c^
**HOMA-β^a^**	-0.401	0.000^c^
**TC^a^, mmol/L**	-0.027	0.802
**TG^a^, mmol/L**	0.103	0.337
**LDL^a^, mmol/L**	0.022	0.837
**HDL^a^, mmol/L**	0.043	0.686
**ApoA1^a^, g/L**	0.106	0.321
**ApoBa, g/L**	0.181	0.090
**FFA^a^, mEq/L**	-0.482	0.000^c^
**HBeAg (+) or (-)**	0.154	0.150
**Viral load copies/ml**	0.087	0.419
**Genotype**	0.143	0.022^b^
**Grade of inflammation**	0.210	0.049^b^
**Grade of fibrosis**	0.099	0.354
**Grade of steatosis**	-0.318	0.002^c^
**With or without steatosis**	-0.761	0.330

^a^Abbreviations: BMI, body mass index; ALT, alanine aminotransferase; AST, aspartateaminotransferase; ALP, alkaline phosphatase; LDH, lactate dehydrogenase; γ-GT, γ-glutamyltranspeptidase; FPG, fasting plasma glucose; FINS, fasting insulin; HOMA-IR, homeostasis model assessment of insulin resistance; HOMA-β, homeostasis-model assessment of beta-cell function; TC, total cholesterol; TG, triglyceride; LDL, low-density lipoprotein; HDL, high-density lipoprotein; Apo A1, apoliporotein A1; Apo B, apolipoprotein B; FFA, free fatty acid.

^b^P < 0.05

^c^P < 0.01

### 4.3. Association of Serum Adiponectin With Liver Histology

There was a statistically positive association between serum adiponectin, and grade of inflammation (rs = 0.210, P = 0.049), but there was no association between serum adiponectin, and stage of fibrosis (rs = 0.099, P = 0.354). Moreover, there was a significant negative correlation between serum adiponectin, and grade of steatosis (rs = -0.318, P = 0.002) (Table 3). However, in subjects with steatosis, there were no association between serum adiponectin, and the grade of inflammation (rs = -0.157, P = 0.377), fibrosis (rs = -0.265, P = 0.130) or steatosis (rs = -0.184, P = 0.298) (Table 3).

**Table 3.** Association of Hepatic Adiponectin Immunoreactivity With Demographic, Metabolic, Histological, and Viral Factors in Patients With Chronic HBV

Characteristic (n=89)	rs^a^	P value
**Gender Male/Female, %**	-0.163	0.126
**Age, y**	0.250	0.018^b^
**BMI^a^, kg/m^2^**	0.235	0.027^b^
**ALT^a^, U/L**	0.111	0.300
**AST^a^, U/L**	0.085	0.430
γ-**GT^a^, U/L**	0.303	0.004^c^
**FPG^a^, mmol/L**	0.092	0.389
**Insulin, mU/L**	-0.232	0.028^b^
**C-Peptide, nmol/L**	0.005	0.960
**Adiponectin, ug/ml**	-0.204	0.055
**HOMA-IR^a^**	0.187	0.080
**Grade of inflammation, % with 0/1/2/3/4**	-0.193	0.070
**Grade of fibrosis, % with 0/1/2/3/4**	-0.252	0.017^b^
**Grade of steatosis, % with 0/1/2/3**	0.589	0.000^c^
**Viral load, copies/ml**	-0.093	0.385

^a^Abbreviations: BMI, body mass index; ALT, alanine aminotransferase; AST, aspartateaminotransferase; γ-GT, γ-glutamyltranspeptidase; FPG, fasting plasma glucose; HOMA-IR, homeostasis model assessment of insulin resistance.

^b^P < 0.05

^c^P < 0.01

### 4.4. Hepatic Immunohistochemistry of Adiponectin, and Adipor2

Immunohistochemistry for adiponectin, and adipoR2 (Figures 2 and 3) was performed in liver biopsies in a subgroup of our study patients. Adiponectin protein expression was localized primarily to endothelial cells of portal vessels, and liver sinusoids. The endothelium of hepatic arteries, and portal veins in portal areas had positive findings uniformly. In the sinusoidal endothelial cells there was variable membrane, and cytoplasmic staining. The plasma within the sinusoids had also positive findings in some cases. No hepatocyte or ductal epithelium staining was observed (11, 12). Biopsies of adiponectin staining in CHB patients without steatosis showed pronounced positivity in the endothelium of vessels in the portal tracts, and in endothelial cells of liver sinusoids (Figure 3A) compared to subjects with steatosis (Figure 2A) (2.26 ± 0.67 vs 1.35 ± 0.48; P = 0.000). Adiponectin staining in patients with steatosis showed less positivity, and staining was found only in the endothelium of vessels in the portal tracts. AdipoR2 protein was localized to hepatocytes showing a predominantly cytoplasmic staining pattern. AdipoR2 staining again tended to be more pronounced in liver biopsies of subjects without steatosis (Figure 3B) compared to the subjects with steatosis (Figure 2B) (2.25 ± 0.37 vs. 1.65 ± 0.29; P= 0.048). Hepatic immunoreactivity was scored as grade 1, 2, and 3. In contrast to serum adiponectin, hepatic adiponectin immunoreactivity was not associated with FPG (P = 0.389), HOMA-IR (P = 0.080), and viral load (P = 0.385). Hepatic adiponectin immunoreactivity was significantly associated with age (rs = 0.250, P = 0.018), BMI (rs = 0.235, P = 0.027), γ-GT (rs = 0.303, P = 0.004), insulin (rs = -0.232, P = 0.028), grade of fibrosis (rs = -0.252, P = 0.017), and steatosis (rs = 0.589, P = 0.000), but not serum adiponectin or any other demographic, metabolic or histological characteristic. There was no association between viral load, and hepatic adiponectin immunoreactivity (P = 0.385) (Table 2).

Figure 2.Hepatic Adiponectin, and Adipor2 Immunoreactivity in Biopsies From CHB Patients With Steatosis (Adiponectin, and adipoR2 immunoperoxidase. Original magnification × 400)

**A):**
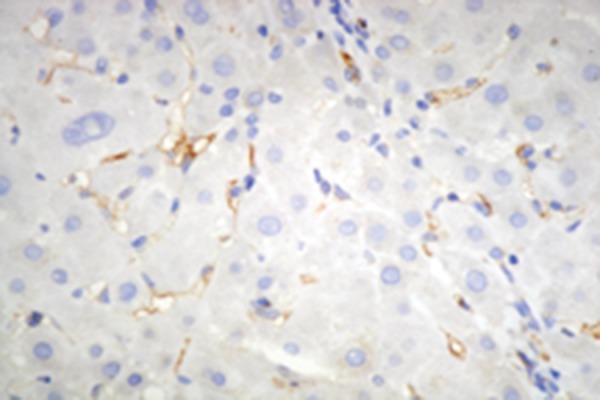
Biopsies show poor staining for adiponectin which was only localized in the endothelium of vessels in the portal tracts.

**B):**
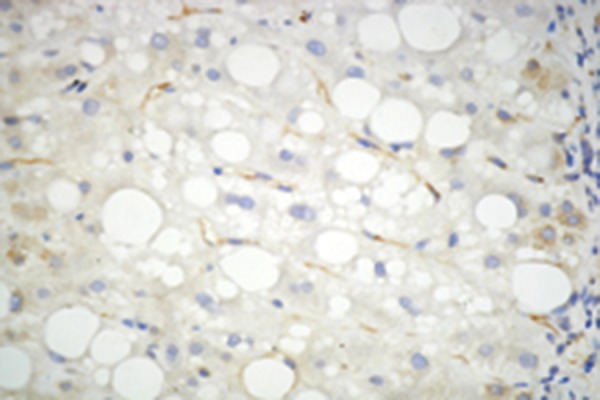
Staining for adipoR2 showed positive staining of parenchymal cells lining the hepatic cells.

Figure 3.Hepatic Adiponectin, and Adipor2 Immunoreactivity in Biopsies From CHB Patients Without Steatosis (Adiponectin, and adipoR2 immunoperoxidase. Original magnification × 400). The arrow shows the expression of adiponectin in liver cell

**A):**
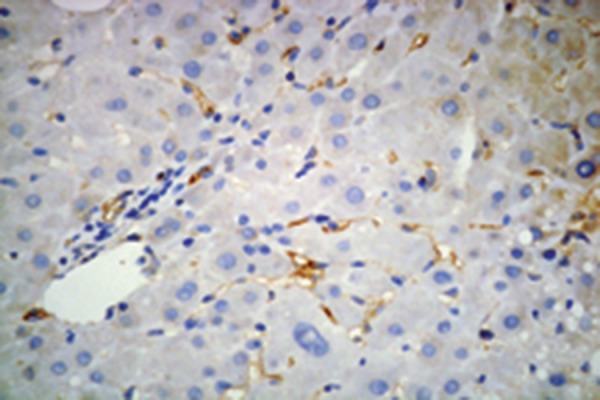
Biopsies showed mild (grade 1) staining for adiponectin with pronounced positivity in the endothelium of vessels in the portal tracts, and in endothelial cells of liver sinusoids.

**B):**
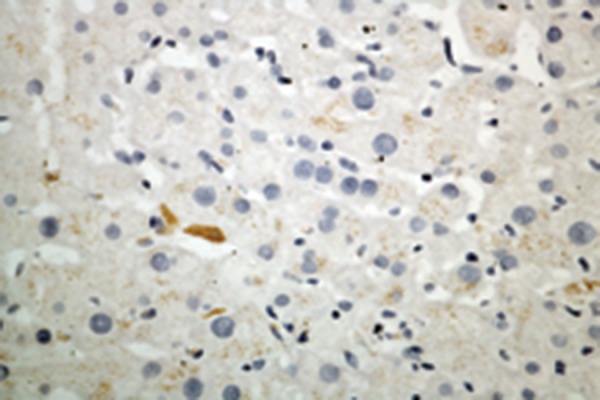
More extensive staining for AdipoR2 was found in parenchymal cells lining the hepatic cell.

### 4.5. Hepatic mRNA Expression of Adiponectin, and Its Receptors

**Figure 4.**
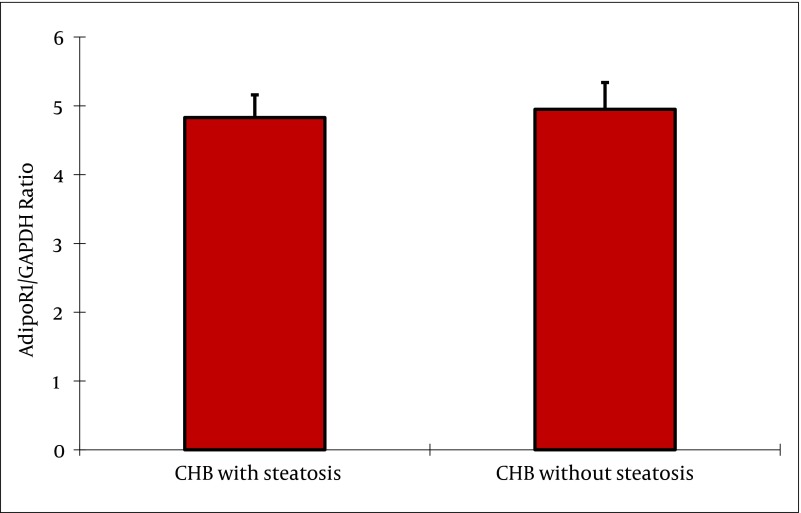
Adiponectin Receptor 1 mRNA Expression Levels in Patients With CHB The expression of adipoR1 mRNA normalized to GAPDH tended to be lower in liver biopsies of subjects with steatosis without reaching statistical significance (4.58 ± 0.37 vs. 4.59 ± 0.47, P = 0.880) compared to the subjects without steatosis.

**Figure 5.**
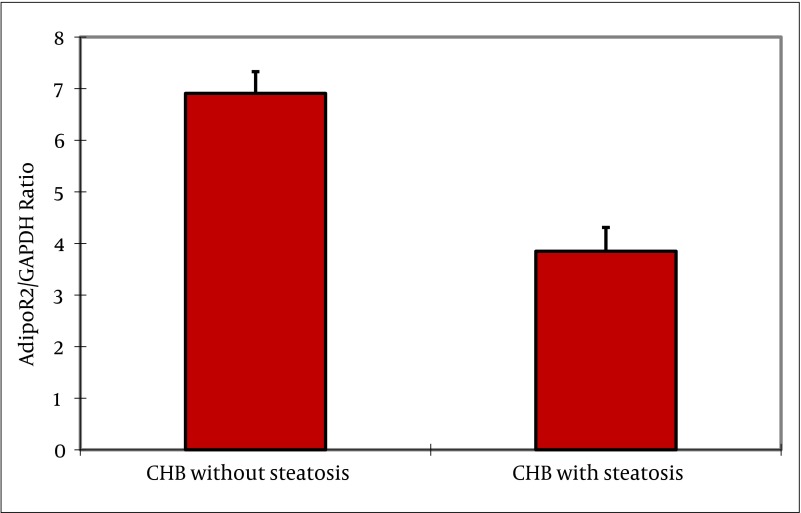
Adiponectin Receptor 2 mRNA Expression Levels in Patients With CHB The expression of adipoR2 mRNA normalized to GAPDH was significantly decreased in liver biopsies of patients with steatosis compared to those without steatosis (3.57 ± 0.33 vs. 7.12 ± 0.67; P = 0.000).

Adiponectin mRNA was not detectable in liver biopsies from patients with chronic HBV up to 45 cycles of amplification, while as a positive control adiponectin mRNA was consistently amplified from human adipose tissue. In contrast, AdipoR1, and AdipoR2 mRNAs were readily detectable in all biopsies examined. In subjects with steatosis, adipoR2 mRNA expression was negatively correlated with BMI (rs = -0.547, P = 0.001), and γ-GT (rs = -0.442, P = 0.009). AdipoR1 mRNA expression was negatively correlated with HOMA-IR (rs = -0.349, P = 0.043), and grade of steatosis (rs = -0.340, P = 0.049). No correlation was found between receptor mRNA expression, and AST and ALT levels. Furthermore, there was no correlation between serum adiponectin, and hepatic adiponectin, adipoR1 or adipoR2 mRNA expression in any group, respectively. As shown in Figure 4, the adipoR1 mRNA expression tended to be lower in liver biopsies of subjects with steatosis without reaching statistical significance (4.58 ±0.37 vs. 4.59 ± 0.47, P = 0.880) compared to subjects without steatosis. As shown in Figure 5, the adipoR2 mRNA expression was significantly decreased in liver biopsies of patients with steatosis compared to those without steatosis (3.57 ± 0.33 vs. 7.12 ± 0.67; P = 0.000). AdipoR1/GAPDH, and adipoR2/GAPDH cDNA ratios are shown in (Figures 4 and 5).

## 5. Discussion

Adiponectin is well recognized as physiologically active polypeptide hormone exclusively derived from mature adipocytes, which plays an important role in diabetes, obesity, atherogenesis, and inflammation. There is much interest in the role of the adipokine, adiponectin, in type 2 Diabetes, cardiovascular disease, and more recently, chronic liver disease. In patients with chronic liver disease due to hepatitis C virus infection, adiponectin was positively correlated with hepatic inflammation, and adiponectin receptors were differentially regulated in the setting of hepatic insulin resistance (12). The aim of the present study was to define the potential role of adipocyte-derived adiponectin, and its receptors, adipoR1 and adipoR2, in the pathogenesis of steatosis in patients with CHB. Recently, CHC with MS was found to be associated with higher insulin resistance, and lower adiponectin level. Adiponectin level, and insulin resistance were significantly correlated (18). We found that serum adiponectin levels were not related togender, and serum ALT level in healthy controls, and serum adiponectin level was similar between healthy adults, and patients with or without steatosis. Our results suggest that serum adiponectin levels were similar in CHB patients with or without steatosis; there were significant negative correlations between serum adiponectin, and BMI, FPG, serum insulin, serum c-peptide, and HOMA-IR. Hepatic adiponectin immunoreactivity was significantly associated with BMI, γ-GT, insulin. In this study we demonstrated that in patients with chronic liver disease due to infection with the hepatitis B virus, adiponectin level, and insulin resistance were significantly negatively correlated. An unexpected finding in this study was a positive association between adiponectin, and hepatic inflammation in patients with CHB, similar to the findings in patients with hepatitis C (12). Adiponectin is reported to have anti-inflammatory properties (7, 8, 10), and supplementation of adiponectin in animal models of hepatic fibrosis attenuated hepatomegaly, steatosis, and inflammation (9). We found that serum adiponectin was significantly associated with the grade of inflammation in patients with chronic hepatitis B (with or without steatosis). Hepatic adiponectin immunoreactivity was significantly associated with the grade of fibrosis, and steatosis. However, serum adiponectin was not associated with the grade of inflammation in subjects with steatosis, and there was a significant correlation between serum adiponectin, and the grade of inflammation in subjects without steatosis. Thus, the identified association between adiponectin, and inflammation in patients with CHB may be reflective of the initiation, and progression of liver disease. In this cohort of patients with chronic hepatitis B, it is possible that the increased adiponectin in relation to hepatic inflammatory activity may be secondary to the response to viral infection. The associations between adiponectin, and the degree of hepatic fibrosis may be disease specific. Julie et al., studied patients with chronic HCV, their results showed that the stage of fibrosis was not related to adiponectin, but the lower levels of serum adiponectin were associated with steatosis only in males (12). Hui and colleagues found that decreased adiponectin was associated with increased grade of steatosis, but not with stage of fibrosis in males, and females with NAFLD/NASH (19). In contrast, a preliminary study reported that adiponectin was associated with stage of fibrosis in patients with biliary liver diseases, and cholestasis (20). In our study, we found that hepatic adiponectin immunoreactivity was negatively correlated with the grade of fibrosis, and positively correlated with steatosis. Notably, adiponectin was mainly localized to endothelial cells of portal vessels, and liver sinusoids, while adipoR2 was exclusively detected in hepatocytes. This may suggest that this hormone/receptor complex could function in a paracrine way in the liver, and this interaction could be impaired in NASH. Kaser found no correlation between circulating adiponectin levels, and liver adiponectin expression (11). This could suggest that liver adiponectin expression is regulated by different factors, such as proinflammatory cytokines such as TNF-α. No association was found between serum adiponectin, and liver adiponectin expression in this cohort. Studies in animals have found increased hepatic adiponectin mRNA expression following toxic injury (21), and Kaser et al found that the levels of expression was very low in liver tissue of patients with NASH (11). The results from Tietge showed that serum adiponectin levels in patients with advanced cirrhosis were significantly elevated (22). We found that the mRNA level of hepatic adiponectin was positively correlated with adipoR2, and there was no correlation between serum adiponectin, and hepatic adiponectin, adipoR1 or adipoR2 expression. No correlation was found between the mRNA level of hepatic adiponectin and AST, ALT levels, and viral load of HBV. Furthermore, there was no correlation between serum adiponectin, and the mRNA levels of hepatic adiponectin, adipoR1 or adipoR2 mRNA. The mRNA level of hepatic adipoR1 was similar in NASH, and simple steatosis. But the levels of hepatic adiponectin, and adipoR2 in patients with NASH were decreased (11). In patients with chronic HCV, hepatic expression of AdipoR1, and AdipoR2 appeared to be differentially regulated in the setting of hepatic insulin resistance, as measured by hepatic PEPCK expression, and in response to serum adiponectin (12). While adipoR2 is predominantly expressed in the liver (5), adipoR1 is mainly expressed in skeletal muscle (5, 6), suggesting that in the liver the effects of adiponectin are predominantly mediated by adipoR2. While adipoR1 is a high affinity receptor for globular adiponectin, adipoR2 can mediate binding of both globular and full-length adiponectin, and thus can increase PPAR-α ligand activity, and fatty acid oxidation by globular, and full-length adiponectin (5). However, we found that in patients with CHB, local effects of adiponectin are limited through two different mechanisms: increased adiponectin mRNA expression, and increased mRNA expression of hepatic adipoR2. While mRNA levels of adipoR1, adipoR2, and adiponectin tended to be lower in liver biopsies of subjects with steatosis compared tosubjects without steatosis, suggesting a pathophysiological role for this adipokine in liver diseases. In patients with chronic HCV, the plasma level of adiponectin inversely correlates with the development of liver steatosis, suggesting that hypoadiponectinemia may contribute to the hepatic steatosis progression, and liver injury. This finding may provide a potential avenue for treating hepatic steatosis in HCV-infected subjects (23). The adiponectin resistance, and sensitivity mediated by AdipoR2 in hepatocytes regulated steatohepatitis progression by changing PPAR-alpha activity, and ROS accumulation, a process in which TGF-beta signaling is implicated. Thus, the liver AdipoR2 signaling pathway could be a promising target in treating NASH (24). Our results suggested that there was no association between several measures of HBV infection, and adiponectin, and its receptors. In conclusion, in patients with chronic HBV, the insulin sensitizing adipokine adiponectin, and its receptor AdipoR2 were associated with steatosis. Adiponectin maybe correlated with inflammation. But adiponectin, and its receptors were not associated with viral factors of HBV. Our results suggest that the role of adiponectin might be impaired in chronic hepatitis B with steatosis.
